# Expression analysis of plant intracellular Ras-group related leucine-rich repeat proteins (PIRLs) in *Arabidopsis thaliana*

**DOI:** 10.1016/j.bbrep.2022.101241

**Published:** 2022-03-05

**Authors:** Md. Firose Hossain, Mst Momtaz Sultana, Ai Tanaka, Amit Kumar Dutta, Takushi Hachiya, Tsuyoshi Nakagawa

**Affiliations:** a Department of Molecular and Functional Genomics, Interdisciplinary Center for Science Research, Shimane University, Matsue, 690-8504, Japan; b Bioresource and Life Sciences, The United Graduate School of Agricultural Sciences, Tottori University, Tottori, 680-8550, Japan; c Department of Agricultural Extension (DAE), Ministry of Agriculture, Dhaka, 1215, Bangladesh; d Department of Microbiology, University of Rajshahi, Rajshahi, 6205, Bangladesh

**Keywords:** *Arabidopsis thaliana*, Leucine-rich repeat, Promoter:GUS assay, Co-expression, Phylogenetic analysis, Pollen

## Abstract

*Arabidopsis thaliana* contains a family of nine genes known as plant intracellular Ras-group related leucine-rich repeat (LRR) proteins (PIRLs). These are structurally similar to animals and fungal LRR proteins and play important roles in developmental pathways. However, to date, no detailed tissue-specific expression analysis of these *PIRLs* has been performed. Therefore, in this study, we generated promoter:GUS transgenic plants for the nine *A. thaliana PIRL* genes and identified their expression patterns in seedlings and floral organs at different developmental stages. Most *PIRL* members showed expression in the root apical region and in the vascular tissue of primary and lateral roots. Shoot apex-specific expression was recorded for *PIRL1* and *PIRL8*. Furthermore, *PIRL1*, *PIRL3, PIRL5*, *PIRL6,* and *PIRL7* showed distinct expression patterns in flowers, especially in pollen and anthers. In addition, co-expression network analysis identified cases where *PIRLs* were co-expressed with other genes known to have specific functions related to growth and development. Taken together, the tissue-specific expression patterns of *PIRL* genes improve our understanding of the functions of this gene family in plant growth and development.

## Highlights

• *PIRL* constituting gene family in *A. thaliana* is widely distributed among plants.
• *PIRL1*, *2*, *3*, and *6* were strongly expressed in anther and/or pollen, consistent with their function in pollen development.
• *PIRL7* was distinctively expressed in pollen and pollen tube, suggesting its role in pollen function.

## 1 Introduction

Leucine-rich repeat (LRR) proteins contain a characteristic LRR domain consisting of 18–29 amino acid repeats rich in leucine residues. These proteins are widespread in plants, animals, and bacteria [1,2]. In animals and lower eukaryotes, an intracellular LRR protein subfamily known as Ras-group LRR proteins with a variably long LRR domain are functionally involved in signal transduction [[3], [4], [5], [6], [7], [8]]. Plants contain a small group of proteins that are structurally analogous to Ras-group LRRs, named plant intracellular Ras-group related LRR proteins (PIRLs) [4,9].

The *Arabidopsis thaliana* genome has nine *PIRL* genes that are categorized into three subfamilies based on their evolutionary relationship [4]. To date, the functions of five PIRLs have been identified. *PIRL6* has been found to be essential for male and female gametogenesis [10], while *PIRL1* and *PIRL9* redundantly work during the differentiation of microspores into pollen [11]. In addition, *PIRL2* and *PIRL3* have been found to be involved in pollen morphogenesis [12]. However, to elucidate the physiological functions of the remaining *PIRL* genes, it is requisite to know their precise expression patterns.

In this study, we used a promoter:GUS assay to examine in detail the expression patterns of *PIRL* genes in *A. thaliana.* We found that *PIRL* genes were expressed in the root, root hairs, shoot apex, leaves, anther, pollen, and pollen tube. We also performed a phylogenetic analysis of *A. thaliana* PIRL proteins with homologous PIRL proteins from other plants and in silico analyses examining the co-expression of *PIRL* genes. The results of these experiments should improve our understanding of the function of *PIRL* genes during plant growth and development.

## 2 Materials and methods

### 2.1 Phylogenetic analysis of PIRLs protein

The amino acid sequences of all nine *A. thaliana* PIRLs were retrieved from The Arabidopsis Information Resource (TAIR) database version 10 (https://www.arabidopsis.org/). A NCBI (https://www.ncbi.nlm.nih.gov/) BLASTP was carried out using *A. thaliana* PIRL1 amino acid sequences as a query to identify PIRL homolog candidates in other plants. A total of 49 amino acid sequences (Supplementary Table 1) were obtained, containing sequences from *Brassica rapa*, *Capsella rubella*, *Oryza sativa*, *Hibiscus syriacus*, *Zea mays*, and *Ricinus communis*. All amino acid sequences were verified by examining their entries in the appropriate databases, e.g., TAIR (https://www.arabidopsis.org/), the Rice Genome Annotation Project (RGAP) (http://rice.plantbiology.msu.edu/), Phytozome v12.1 (https://phytozome.jgi.doe.gov/), and Uniprot (https://www.uniprot.org/). ClustalW was used to perform multiple sequence alignment. A phylogenetic tree was constructed using the neighbor-joining (NJ) method [13] implemented in MEGA7 [14] with 1,000 bootstrap replicates. The domain structures of the nine *A. thaliana* PIRL proteins were predicted using Uniprot and were drawn using the MyDomains-Image Creator (https://prosite.expasy.org/mydomains/).

### 2.2 Plasmid construction

The promoter entry clones pDONR201-ProPIRL1 to pDONR201-ProPIRL9 were prepared with the primers listed in Supplementary Table 2. The promoter:GUS binary constructs were prepared with pDD333 and pGWB3450 vectors according to the method described by Sultana et al. [15].

### 2.3 Generation of transgenic A. thaliana

Transformation of *Agrobacterium tumefaciens* C58C1 (pMP90) was performed using the freeze-thaw method as per Weigel and Glazebrook [16]. Transformation of *A. thaliana* (Col-0 accession) was performed using the floral inoculation method [17]. Harvested T0 seeds were kept at 4 °C for 3 days, after which they were grown on Murashige and Skoog (MS) agar medium containing kanamycin (30 mg/L) and Cefotax (100 mg/L) (Chugai Pharmaceutical Co., Tokyo, Japan) at 22 °C under continuous light. Fifteen-day-old selected plants (T1) were transferred to Jiffy-7 plant pots (Jiffy Products International BV, Zwijndrecht, Belgium) and were grown at 22 °C under a 16-hr light/8-hr dark daily cycle.

### 2.4 Histochemical GUS staining

Plants were selected at different time points for histochemical staining, including 1-day-after-germination (DAG) seedlings, as well as plants at 4-DAG, 8-DAG, 16-DAG, and the flowers of 40-DAG plants. Staining was performed as described by Nakamura et al. [18] and plants were observed using a stereo microscope (SZX16, Olympus, Tokyo, Japan) equipped with a Nikon digital camera (DXM1200F) as well as an all-in-one microscope (BZ-710, Keyence, Osaka, Japan).

### 2.5 In silico expression and co-expression of PIRL genes

The expression value was obtained for each *PIRL* gene in several different tissues (cotyledon, hypocotyl, root, leaf, flower, sepal, petal, and pollen) using microarray data from the Arabidopsis eFP browser [19]. Expression values were then normalized using the log2 transformation. Finally, a heat map was generated by TBtools software [20]. Microarray data showing visual expression during different developmental stages was also retrieved from the Arabidopsis eFP browser [19]. The co-expression gene network of *PIRLs* and co-expressed genes were analyzed using the ATTED-II (https://atted.jp/) [21].

## 3 Results

### 3.1 Phylogenetic analysis shows a relationship between A. thaliana PIRLs and PIRLs found in other species

Phylogenetic analysis of amino acid sequences from *A. thaliana* (PIRL), *Brassica rapa* (BrPIRL), *Capsella rubella* (CrPIRL), *Oryza sativa* (OsPIRL), *Hibiscus syriacus* (HsPIRL)*, Zea mays* (ZmPIRL), *and Ricinus communis* (RcPIRL) showed that, the PIRLs from *A. thaliana* showed some similarity to those from other plants. Our tree revealed that the phylogenetic relationships between and among species were clustered into six groups (Fig. 1A). In group I, PIRL4 and PIRL5 formed a cluster with CrPIRL4 and CrPIRL5, with bootstrap values of 93 and 87, respectively. The highest number of clusters was found in group III, where PIRL6 formed a cluster with BrPIRL6, and PIRL7 and PIRL8 formed a cluster with CrPIRL7 and CrPIRL8, respectively. Group IV contained PIRL2 and PIRL3, which formed a cluster with CrPIRL2 and CrPIRL3. Finally, PIRL1 and PIRL9 formed a cluster with BrPIRL1 and CrPIRL9, and these were grouped together into Group V. Next, we predicted the number of LRR in each *A. thaliana* PIRL (Fig. 1B and Supplementary Table 3). All PIRLs were found to contain the defining LRR; PIRL4 and PIRL5 contained the highest number (11) of LRRs, while PIRL1, PIRL2, PIRL3, PIRL6, PIRL7, PIRL8, and PIRL9 all showed 10 LRRs (Fig. 1B and Supplementary Table 3).

**Fig. 1:**
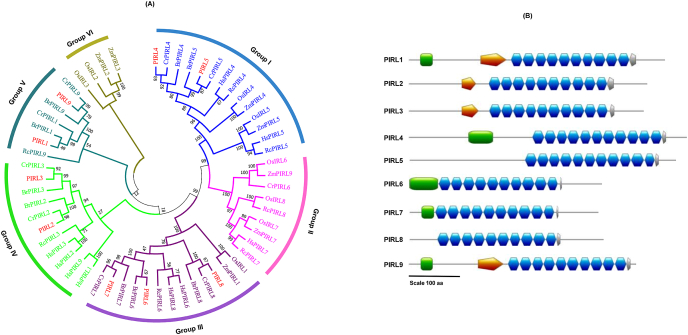
**Phylogenetic tree of *A. thaliana* PIRLs including homologous PIRLs from other plants as well as the domain structure of the nine *A. thaliana* PIRLs**. (A) An unrooted phylogenetic tree was created using the full-length protein sequences of the nine *A. thaliana* PIRLs and their homologs found in other plants. Group I, II, III, IV, V, and VI are denoted by blue, fuchsia, purple, lime, teal, and olive, respectively, while PIRLs from *A. thaliana* are indicated in red. The prefixes Br, Cr, Os, Hs, Zm, and Rc were used to designate *Brassica rapa*, *Capsella rubella*, *Oryza sativa*, *Hibiscus syriacus*, *Zea mays*, and *Ricinus communis*, respectively. BrPIRLs, CrPIRLs, HsPIRLs, ZmPIRLs, and RcPIRLs were numbered according to their registered names in the NCBI database. (B) Domain structure of the nine *A. thaliana* PIRLs. The following scheme was used to indicate domains: green squares represent disordered regions; orange horizontal pentagons indicate coiled coil regions; blue hexagons indicate LRR; gray horizontal pentagons indicate the GVYW motif.

### *3.2* *PIRL* expression in different vegetative tissues in *A. thaliana*

**Fig. 2:**
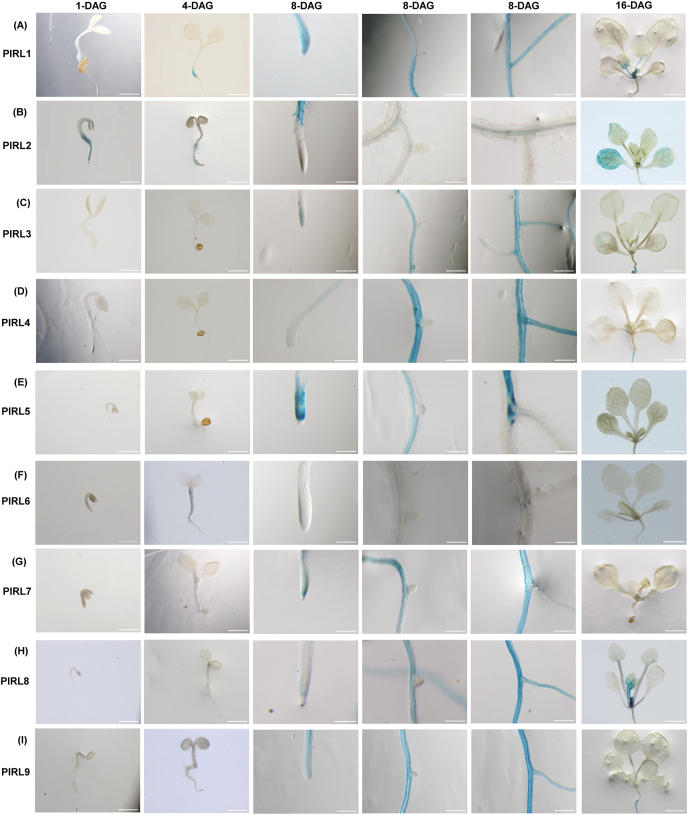
**Expression patterns of *PIRL* genes in the vegetative tissues of *A. thaliana*.** (A) ProPIRL1:GUS, (B) ProPIRL2:GUS, (C) ProPIRL3:GUS, (D) ProPIRL4:GUS, (E) ProPIRL5:GUS, (F) ProPIRL6:GUS, (G) ProPIRL7:GUS, (H) ProPIRL8:GUS, (I) ProPIRL9:GUS. GUS staining is shown for 1-DAG seedlings, 4-DAG seedlings, 8-DAG primary root tips (left), 8-DAG initiated lateral roots (center), 8-DAG differentiated roots (right), and 16-DAG aboveground organs of transgenic *A. thaliana*. Scale bars = 1 mm.

We subjected T2 or T3 plants from ProPIRL:GUS transgenic lines to histochemical GUS staining at different developmental stages. As shown in Fig. 2, seedlings (1-, 4-, 8-, and 16-DAG) exhibited different expression patterns in their vegetative organs. For *PIRL1*, GUS expression was observed at the junction between the root and hypocotyl at 4-DAG, at the primary root tip and in the differentiated region of the primary and secondary roots at 8-DAG, and in the shoot apex at 16-DAG (Fig. 2A). For *PIRL2*, we observed unique expression in root hair, and while lower expression was observed in the vascular tissues of the primary and secondary roots from 1- to 8-DAG, we also observed moderate expression in leaves and strong expression in stipules at 16-DAG (Fig. 2B). *PIRL3*, *PIRL4*, *PIRL7*, and *PIRL9* showed two similar trends: for each of these *PIRL* genes, we observed GUS expression in root (1- to 8-DAG) but faint or no signal in aboveground organs at 16-DAG (Fig. 2C, D, G and I). For *PIRL5*, we observed expression in the root tip and in the vascular tissue of the first and secondary roots at 4- and 8-DAG, but again almost no expression in aboveground organs at 16-DAG (Fig. 2E). *PIRL8* showed GUS expression in roots (8-DAG), the shoot apex region, and young leaves (Fig. 2H). We did not observe considerable expression of *PIRL6* in any vegetative organs (Fig. 2F). All the results of GUS staining experiments are summarized in Supplementary Table 4.

### *3.3* *PIRLs* are expressed in various reproductive tissues in *A. thaliana*

**Fig. 3:**
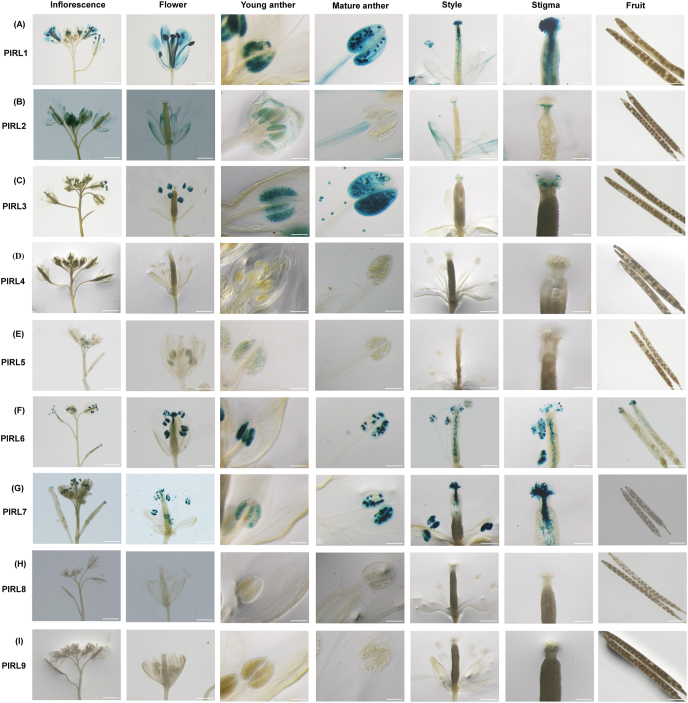
**Expression patterns of *PIRL* genes in the floral organs of *A. thaliana*.** (A) ProPIRL1:GUS, (B) ProPIRL2:GUS, (C) ProPIRL3:GUS, (D) ProPIRL4:GUS, (E) ProPIRL5:GUS, (F) ProPIRL6:GUS, (G) ProPIRL7:GUS, (H) ProPIRL8:GUS, (I) ProPIRL9:GUS. GUS staining is shown for the inflorescence, flower, young anther, mature anther, style, stigma, and fruit tissue of 40-DAG transgenic *A. thaliana*. Scale bars = 1 mm.

We subjected the flowers of 40-DAG transgenic plants to GUS staining. For *PIRL1*, GUS was weakly expressed in petals and filaments, but was strongly expressed in anthers and pollen (Fig. 3A). Expression was also detected in the pollen tube (Fig. 3A). For *PIRL2*, expression was strong in flower buds but was lower in sepals, petals, filaments, and stigmas of open flowers (Fig. 3B). We observed distinct expression of *PIRL3* in anthers and pollen, but not in the pollen tube (Fig. 3C). Next, for *PIRL4*, we did not find any expression in any floral organs whatsoever (Fig. 3D). Similarly, *PIRL5* was detected very weakly and only in anthers (Fig. 3E). However, clear GUS expression was detected in both pollen and pollen tubes for both *PIRL*6 and *PIRL*7 (Fig. 3F and G). GUS activity was not observed in any floral organ for either *PIRL8* or *PIRL9* (Fig. 3H and I). All GUS staining results are summarized in Supplementary Table 4.

### 3.4 In silico expression and co-expression analysis of PIRLs

**Fig. 4:**
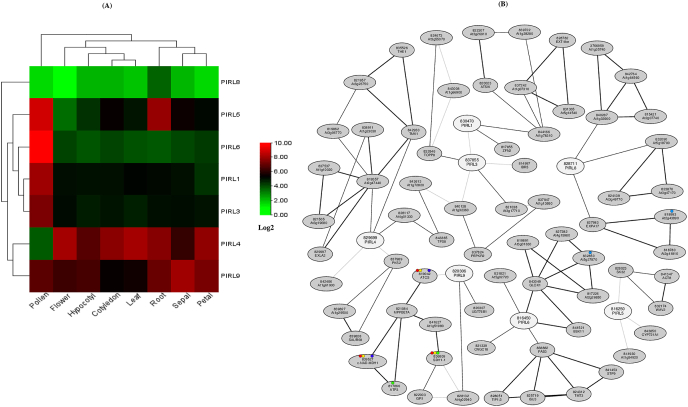
**In silico gene expression and co-expression analyses of *PIRL* genes.** (A) Gene expression (Log2 transformed) heat map of *A. thaliana PIRL* genes at different developmental stages. *PIRLs* are shown on the right side, and tissues are indicated at the bottom. Red indicates a higher level of gene expression, while green indicates lower expression. (B) Co-expression networks were generated by the ATTED-II using *PIRL* as query genes. Red, yellow, blue, sky-blue, and green circles indicate groups of genes involved in different metabolic pathways.

We generated a heat map that used microarray datasets of *PIRL* genes sourced from the Arabidopsis eFP browser (Fig. 4A). This heat map analysis showed that *PIRL6* was specifically expressed in pollen (Fig. 4A), that *PIRL1* and *PIRL3* were expressed strongly in pollen and moderately in vegetative organs (Fig. 4A), and that *PIRL5* was expressed in pollen and roots (Fig. 4A). This analysis also showed that both *PIRL4* and *PIRL9* were expressed in a variety of organs, although *PIRL4* was not expressed in pollen (Fig. 4A). Interestingly, weak expression in root tissue was observed for *PIRL8* (Fig. 4A). Moreover, a microarray study using visual expression of *PIRL* genes during developmental stages revealed that *PIRL1* and *PIRL3* were significantly expressed in floral organs, specifically in mature pollen rather than vegetative organs (Supplementary Fig. S1). According to the microarray expression developmental map, *PIRL4* and *PIRL9* were expressed in a number of regions, while no expression was found for *PIRL4* in pollen (Supplementary Fig. S1). *PIRL5* and *PIRL6* showed vigorous expression in mature pollen, whereas root-specific expression was also observed for *PIRL4* and *PIRL8,* respectively. The microarray datasets contained no expression values for *PIRL2* and *PIRL7*. Further, to identify the functional relationship between *PIRLs* and other genes, we generated a co-expression network using the ATTED-II (ath-m.c9-0 platform) (Fig. 4B). Of the nine *PIRL* genes, only *PIRL1* and *PIRL3* were found to co-express with each other. Furthermore, *PIRL1* expression was correlated with the expression of VQ-motif containing protein 9 (VQ9, At1g78310), which is a class of transcription factor [22], and zinc finger nuclease 2 (ZFN2, AT2G32930) (Fig. 4B). We also found that *PIRL3* co-expressed strongly with a member of the Type One Protein Phosphatase family known as TOPP8 (AT5G27840), as well as IBR5 (AT2G04550) and an F-box domain-containing protein (At3g17710) (Fig. 4B). Expression of *PIRL4* was found to strongly correlate with expression of TMK1 (AT1G66150) (Fig. 4B), while *PIRL5* was weakly co-expressed with SK32 (AT4G00720) and CYP721A1 (AT1G75130) (Fig. 4B). *PIRL6* was co-expressed with GLOX1 (AT1G67290) and PAB3 (AT1G22760) (Fig. 4B), two genes whose expressions are known to have been restricted to the anther and tapetum [23]. *PIRL8* showed co-expression with IMMUNE ASSOCIATED NUCLEOTIDE BINDING 4 (IAN4, At1g33900) and a member of the Alpha Expansion gene family (EXPA17, AT4G01630) (Fig. 4B). The expression of *EXPA17* is root-specific and it is vital for lateral root formation [24]. Finally, *PIRL9* was found to co-express with UDP-DEPENDENT GLYCOSYLTRANSFERASE 76B1 (UGT76B1, AT3G11340) and At4g02940 (Fig. 4B). Co-expression data was not available for *PIRL2* and *PIRL7.*

## 4 Discussion

LRR proteins are found in many plant species, where they perform important biological functions linked to growth and development. For instance, many proteins from the LRR-receptor-like kinases (RLKs) and related-receptor-like proteins (RLPs) families play crucial roles in plant growth and development and in defense-related pathways [[25], [26], [27]]. In *A. thaliana*, nine *PIRLs* have been identified, some of which have been previously examined for functional roles in developmental signaling. In the present study, we used promoter:GUS assays and in silico approaches to analyze the amino acid sequences and detailed expression patterns of nine *PIRLs* found in *A. thaliana*. Phylogenetic analysis revealed the relationships of the *A. thaliana* PIRL proteins with other plants PIRLs and resolved all sequences into six groups (Fig. 1A). Each *A. thaliana* PIRL was found to be close in sequence to one of the PIRLs found in *C. rubella* (CrPIRL) and others from *B. rapa* (BrPIRL). *A. thaliana* PIRL1 and PIRL9 belong to Group V, a group that also contains PIRLs from *C. rubella* and *B. rapa*. *A. thaliana* PIRL4 and PIRL5 belong to a clade that includes PIRL4 and PIRL5 from both *C. rubella* and *B. rapa*, designated here as Group I. This group also contained PIRL4 and PIRL5 from *O. sativa*, *Z. mays*, *H. syriacus*, and *R. communis*. PIRL6, PIRL7, and PIRL8 from *A. thaliana*, *C. rubella* and *B. rapa* all belong to Group III, a group that also includes PIRL1 from *O. sativa* and *Z. mays*. PIRL2 and PIRL3 from *A. thaliana*, *C. rubella*, and *B. rapa* belong to the same clade, designated here as Group IV. Interestingly, Groups IV and V did not include PIRLs found in *O. sativa* and *Z. mays.* Group VI contains PIRLs only from rice and maize. Focusing on *A. thaliana* and rice, there is no *OsIRL* corresponding to *PIRL1*, *PIRL9*, *PIRL2* and *PIRL3*, and there is no *PIRL* corresponding to *OsIRL2* and *OsIRL3*. *PIRL1* and *PIRL9* have been reported to be essential for pollen development [11]. Although the function of *OsIRL2* and *OsIRL3* is not yet known, they may be redundantly involved in pollen development like *PIRL1* and *PIRL9* because they are most similar to *PIRL1* and *PIRL9* among *OsIRLs*, and their expression was shown in stamens [9]. *OsIRL2* and *OsIRL3* may have diverged and acquired additional functions in rice. For example, expression of *OsIRL2* and *OsIRL3* is also shown in endosperm [9]. Since the *osirl3* mutant exhibited a normal phenotype, analysis of *osirl2*;*osirl3* double mutant will be a clue to clarity their function. Analysis of the domain organizations by Uniprot showed that the number of LRRs present in *A. thaliana* PIRLs is 10 or 11 (Fig. 1B and Supplementary Table 3). The number of LRRs found in PIRL1, PIRL2, PIRL3, PIRL6, PIRL7, PIRL8, and PIRL9 was one more than reported by Forsthoefel et al. [4]. This difference may be due to differences in the length of the first LRR between the previous prediction and the Uniprot prediction used in this study. Our expression experiments revealed that *PIRL1*, *PIRL3*, and *PIRL6* were preferentially expressed in pollen (Fig. 4A). Microarray developmental map, RNA-seq [19,28] and promoter:GUS assays (Fig. 3 and Supplementary Fig. S1) also indicated strong expression of *PIRL1*, *PIRL3*, and *PIRL6* in pollen. These results are consistent with reports that have demonstrated that *PIRL1* and *PIRL9* are vital for the differentiation of microspores into pollen [11]. In addition, recent studies have also shown that *pirl2;pirl3* double mutant generated a wide range of abnormal pollen morphologies [12]. Moreover, our promoter:GUS assay of *PIRL6* showed expression also in the ovule (Fig. 3F), a finding that is consistent with a previous result that showed that *PIRL*6 was essential for both male and female gametogenesis processes in *A. thaliana* [10]. Forsthoefel et al. [10] reported abundant functional transcript in flowers and a small amount of non-functional transcript by alternative splicing in leaf and root. These and our promoter:GUS results indicated transcriptional and post-transcriptional regulation of *PIRL6*. Next, our co-expression analysis showed that the expression of *PIRL1* and *PIRL3* are correlated with each other and that *PIRL1* expression is also associated with the expression of *VQ9* and *ZFN2* (Fig. 4B). The essential role of VQ proteins in vegetative plant growth, differentiation, and seed development has been previously described [22]. The relative expression of *VQ9* has also been reported in roots by Hu et al. [29], a finding that is consistent with the observed expression of *PIRL1* in roots. Taken together, these findings suggest a contribution of *PIRL1* to root development in addition to its role in pollen formation. *PIRL1* and *PIL9* are closely related gene pairs, and *pirl1;pirl9* double knockout plants (*pirl1* −/−, *pirl9* −/−) were not established due to the developmental defect of *pirl1;pirl9* microspore (*pirl1* -, *pirl9* -) into viable pollen [11]. Analysis of double mutants using weaker alleles may reveal their function in the development of vegetative organs, including roots. Next, we also identified *TOPP8* and *IBR5* as genes that were co-expressed with *PIRL3*. *TOPP8* is also known as *ATUNIS2* (*AUN2*) and plays a vital role in pollen germination and pollen tube growth [30]. Pollen- and root-specific expression of *AUN2* has also been reported by Franck et al. [30], who used a microarray analysis that showed a pattern of expression resembling that of *PIRL3*, further suggesting their functional similarity. We also found that *PIRL6* expression was strongly correlated with the expression of genes coding for glyoxal-oxidase-related protein (GLOX1) and PAB3. *GLOX1* was reported to be important for pollen development; Phan et al. [23] found *GLOX1* promoter-driven GUS expression within anthers of floral buds as well as strong GUS activity in mature and released pollen grains. Belostotsky [31] also described tapetum and pollen-specific expression of *PAB3* after performing promoter:GUS assays in *A. thaliana.* These results support the possibility that the function of *PIRL6* in pollen development is related to *GLOX1* and *PAB3*. Although no expression values were available in the database for *PIRL7*, our promoter:GUS assay revealed clear expression of *PIRL7* both in pollen and in the pollen tube (Fig. 3G), suggesting that *PIRL7* plays a role in pollen development. The promoter:GUS assay of *PIRL2* showed unambiguous staining of filaments (Fig. 3B). Therefore, the requirement of *PIRL2* for pollen development [12] may be exerted in the filament. We obtained root-specific promoter:GUS expression for *PIRL4* (Fig. 2D). As shown in Fig. 4, co-expression analysis revealed that *PIRL4* was co-expressed with LRR-RLK TMK1 [32], which has been found to be expressed in roots by RT-PCR and promoter:GUS assays [33]. Dai et al. [33] also reported that the root lengths of *tmk1; tmk4* double mutants were approximately one-third of that of the wild type. The biological functions of *PIRL4* remain unknown; however, co-expression of *PIRL4* and *TMK1* in roots suggests that *PIRL4* may be involved in the growth and development of roots. Furthermore, we also observed promoter:GUS expression of *PIRL5*, *PIRL8*, and *PIRL9* in the root apical region, as well as in the primary and secondary roots (Fig. 2E, H, and I). According to the heat map and microarray developmental map, *PIRL5*, *PIRL8*, and *PIRL9* are also known to be expressed in roots (Fig. 4A and Supplementary Fig. S1). In addition, we found that *PIRL8* showed co-expression with *EXPA17*; a previous study by Lee and Kim [24], who used the promoter:GUS of *EXPA17* and recorded its expression in lateral root and lateral root primordia. Therefore, they suggested that *EXPA17* may be required to promote lateral root formation during auxin response. Together, these findings signify that *PIRL8* may have root-specific functions.

## 5 Conclusion

In this study, we investigated the detailed expression patterns of nine *A. thaliana PIRL* genes at different developmental stages. Promoter:GUS assay showed that most *PIRLs* were expressed in roots and in the vasculature of the primary and lateral roots. In addition, we observed that some *PIRLs* were strongly expressed in floral organs, suggesting their possible biological function to regulate the growth and development of these organs. Moreover, co-expression network analysis indicated that functionally similar *PIRLs* are expressed simultaneously. Therefore, these results will be helpful for future tissue-specific expression analyses of other LRR proteins and may provide useful information to improve our understanding of their biological function.

## Author contribution statement

MFH, AT, AKD and TN conceived the project and designed the experiments. MFH, MMS, AT, TH, and TN performed experiments and conducted data analysis. MFH, TH, and TN wrote the manuscript. All authors read and approved the final manuscript.

## Declaration of competing interest

The authors declare that this study was conducted in the absence of any commercial relationships that could lead to any potential conflicts of interest.

## Figures and Tables

**figs1:**
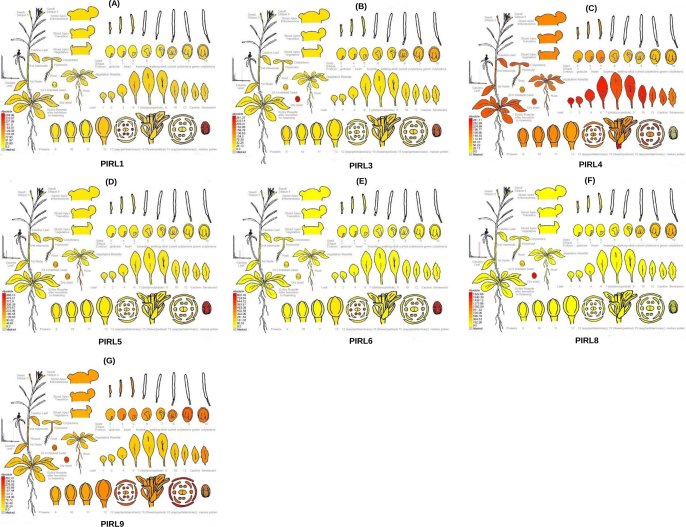


## Data Availability

Data will be made available on request.
