# Supplementary material for: Expression analysis of plant intracellular Ras-group related leucine-rich repeat proteins (PIRLs) in Arabidopsis thaliana
Source: Biochem Biophys Rep. 2022 Mar 5;30:101241. doi: 10.1016/j.bbrep.2022.101241 (PMC8904235; doi:10.1016/j.bbrep.2022.101241)
Supplement: Multimedia component 2 [file mmc2.docx]

**Supplementary Table 2.**  Synthetic Oligonucleotides

| **Oligonucleotide Name*** | **Sequence**** |
| --- | --- |
| ProPIRL1-F (-966) | AAAAAGCAGGCTCCGACACTTCACACGCAGACCATTTCTC |
| ProPIRL1-R (+3) | AGAAAGCTGGGTAGACACTCATTGTTGGGAGCTTGATGG |
| ProPIRL2-F (-2020) | AAAAAGCAGGCTCCGACACTGAATCCGTGTTATGGTTGTTAT |
| ProPIRL2-R (+3) | AGAAAGCTGGGTAGACACTCATGGTTTTTGTGATTATTAGTC |
| ProPIRL3-F (-921) | AAAAAGCAGGCTCCGACACTATTCTCCGGCAAGAAACCTT |
| ProPIRL3-R (+3) | AGAAAGCTGGGTAGACACTCATTCTCAATGAGATTGAAGATGAGAGC |
| ProPIRL4-F (-1080) | AAAAAGCAGGCTCCGACACTCACATGGCGTTTTCCTCTCT |
| ProPIRL4-R (+3) | AGAAAGCTGGGTAGACACTCATTACAAAATTTGGAAGAAACGAAAAG |
| ProPIRL5-F (-2198) | AAAAAGCAGGCTCCGACACTGCTCTTTGGTTACAGGGATG |
| ProPIRL5-R (+3) | AGAAAGCTGGGTAGACACTCATTTTCTACAAAATCAAAATTCAC |
| ProPIRL6-F (-2105) | AAAAAGCAGGCTCCGACACTACAGAACACCTGACATTAACTA |
| ProPIRL6-R (+3) | AGAAAGCTGGGTAGACACTCATCTTTCACACTATCTATATATA |
| ProPIRL7-F (-1999) | AAAAAGCAGGCTCCGACACTCCTATTTATCATGAAATTTGGGT |
| ProPIRL7-R (+3) | AGAAAGCTGGGTAGACACTCATCGTTTTATGTGTGTTTGTAT |
| ProPIRL8-F (-2098) | AAAAAGCAGGCTCCGACACTCGTAGTACTATGTTTTTCAGCT |
| ProPIRL8-R (+3) | AGAAAGCTGGGTAGACACTCATATCTTCCTATGTGTATGTG |
| ProPIRL9-F (-2073) | AAAAAGCAGGCTCCGACACTGGGAAGAGAAAGATCACTGG |
| ProPIRL9-R (+3) | AGAAAGCTGGGTAGACACTCATTGTTTTCGTTAAGCTTCGG |

*The A of the translational initiation codon is designated +1.

**Underlines indicate regions corresponding to partial *att*B1 and *att*B2 sequences (12 bp).
