# Supplementary material for: Expression analysis of plant intracellular Ras-group related leucine-rich repeat proteins (PIRLs) in Arabidopsis thaliana
Source: Biochem Biophys Rep. 2022 Mar 5;30:101241. doi: 10.1016/j.bbrep.2022.101241 (PMC8904235; doi:10.1016/j.bbrep.2022.101241)
Supplement: Multimedia component 3 [file mmc3.docx]

**Supplementary Table 3.** Predicted domain of PIRLs in *A. thaliana*

| **Gene name** | **Protein length (aa)** | **Domain name** | **Position of domain (aa)** |
| --- | --- | --- | --- |
| PIRL1 | 506 | LRR1 | 203-225 |
|  |  | LRR2 | 226-249 |
|  |  | LRR3 | 251-272 |
|  |  | LRR4 | 273-295 |
|  |  | LRR5 | 297-319 |
|  |  | LRR6 | 320-342 |
|  |  | LRR7 | 344-364 |
|  |  | LRR8 | 365-389 |
|  |  | LRR9 | 390-412 |
|  |  | LRR10 | 414-436 |
|  |  | Region (Disordered) | 24-48 |
|  |  | Coiled coil | 143-193 |
|  |  | GVYW motif | 437-449 |
| PIRL2 | 471 | LRR1 | 159-182 |
|  |  | LRR2 | 183-205 |
|  |  | LRR3 | 206-229 |
|  |  | LRR4 | 231-251 |
|  |  | LRR5 | 253-275 |
|  |  | LRR6 | 276-298 |
|  |  | LRR7 | 300-321 |
|  |  | LRR8 | 324-346 |
|  |  | LRR9 | 347-369 |
|  |  | LRR10 | 371-392 |
|  |  | Coiled coil | 106-133 |
|  |  | GVYW motif | 393-405 |
| PIRL3 | 464 | LRR1 | 160-183 |
|  |  | LRR2 | 184-206 |
|  |  | LRR3 | 207-230 |
|  |  | LRR4 | 232-252 |
|  |  | LRR5 | 254-275 |
|  |  | LRR6 | 276-299 |
|  |  | LRR7 | 301-322 |
|  |  | LRR8 | 323-347 |
|  |  | LRR9 | 348-370 |
|  |  | LRR10 | 372-393 |
|  |  | Coiled coil | 106-138 |
|  |  | GVYW motif | 398-406 |
| PIRL4  PIRL4 | 549  549 | LRR1 | 245-268 |
|  |  | LRR2 | 269-291 |
|  |  | LRR3 | 293-313 |
|  |  | LRR4 | 314-337 |
|  |  | LRR5 | 339-360 |
|  |  | LRR6 | 362-383 |
|  |  | LRR7 | 384-406 |
|  |  | LRR8 | 407-430 |
|  |  | LRR9 | 432-454 |
|  |  | LRR10 | 455-476 |
|  |  | LRR11 | 478-500 |
|  |  | Region (Disordered) | 119-167 |
|  |  | GVYW motif | 501-508 |
| PIRL5 | 526 | LRR1 | 229-252 |
|  |  | LRR2 | 253-275 |
|  |  | LRR3 | 276-297 |
|  |  | LRR4 | 298-321 |
|  |  | LRR5 | 323-344 |
|  |  | LRR6 | 346-367 |
|  |  | LRR7 | 368-390 |
|  |  | LRR8 | 391-414 |
|  |  | LRR9 | 416-437 |
|  |  | LRR10 | 438-463 |
|  |  | LRR11 | 465-484 |
|  |  | GVYW motif | 485-492 |
| PIRL6 | 380 | LRR1 | 59-82 |
|  |  | LRR2 | 83-105 |
|  |  | LRR3 | 107-129 |
|  |  | LRR4 | 130-152 |
|  |  | LRR5 | 154-176 |
|  |  | LRR6 | 177-199 |
|  |  | LRR7 | 201-222 |
|  |  | LRR8 | 223-247 |
|  |  | LRR9 | 248-271 |
|  |  | LRR10 | 273-293 |
|  |  | Region (Disordered) | 1-57 |
|  |  | GVYW motif | 298-301 |
| PIRL7 | 373 | LRR1 | 52-75 |
|  |  | LRR2 | 76-98 |
|  |  | LRR3 | 100-122 |
|  |  | LRR4 | 123-145 |
|  |  | LRR5 | 147-169 |
|  |  | LRR6 | 170-192 |
|  |  | LRR7 | 194-215 |
|  |  | LRR8 | 216-240 |
|  |  | LRR9 | 241-264 |
|  |  | LRR10 | 266-286 |
|  |  | Region (Disordered) | 25-49 |
|  |  | GVYW motif | 291-294 |
| PIRL8 | 383 | LRR1 | 56-79 |
|  |  | LRR2 | 80-102 |
|  |  | LRR3 | 104-126 |
|  |  | LRR4 | 127-149 |
|  |  | LRR5 | 151-173 |
|  |  | LRR6 | 174-197 |
|  |  | LRR7 | 199-219 |
|  |  | LRR8 | 221-244 |
|  |  | LRR9 | 245-268 |
|  |  | LRR10 | 270-290 |
|  |  | GVYW motif | 291-298 |
| PIRL9 | 449 | LRR1 | 197-219 |
|  |  | LRR2 | 220-243 |
|  |  | LRR3 | 245-266 |
|  |  | LRR4 | 267-289 |
|  |  | LRR5 | 291-312 |
|  |  | LRR6 | 313-336 |
|  |  | LRR7 | 338-359 |
|  |  | LRR8 | 360-383 |
|  |  | LRR9 | 384-406 |
|  |  | LRR10 | 408-430 |
|  |  | Region (Disordered) | 25-48 |
|  |  | Coiled coil | 138-187 |
|  |  | GVYW motif | 431-438 |
