# Supplementary material for: Expression analysis of plant intracellular Ras-group related leucine-rich repeat proteins (PIRLs) in Arabidopsis thaliana
Source: Biochem Biophys Rep. 2022 Mar 5;30:101241. doi: 10.1016/j.bbrep.2022.101241 (PMC8904235; doi:10.1016/j.bbrep.2022.101241)
Supplement: Multimedia component 4 [file mmc4.docx]

**Supplementary Table 4.** Summarized result of promoter:GUS assay of *PIRL*s in *A. thaliana*

|  | Root | | |  | Shoot | | | |  | Flower | | | | | | | |
| --- | --- | --- | --- | --- | --- | --- | --- | --- | --- | --- | --- | --- | --- | --- | --- | --- | --- |
|  | Primary root tip | Root hair | Differentiated root |  | Shoot apex | Stipule | Young leaf | Mature leaf |  | Sepal | Petal | Filament | Anther | Pollen | Pollen tube | Stigma | Ovule |
| *PIRL1* | ++ | ̶ | ++ |  | ++ | ̶ | + | ̶ |  | + | + | + | ++ | ++ | ++ | ++ | ̶ |
| *PIRL2* | ̶ | ++ | + |  | ̶ | ++ | ̶ | ++ |  | + | + | + | ++ | ̶ | ̶ | + | ̶ |
| *PIRL3* | + | ̶ | ++ |  | ̶ | ̶ | ̶ | ̶ |  | ̶ | ̶ | ̶ | ++ | ++ | ̶ | + | ̶ |
| *PIRL4* | ++ | ̶ | ++ |  | ̶ | ̶ | ̶ | ̶ |  | ̶ | ̶ | ̶ | ̶ | ̶ | ̶ | ̶ | ̶ |
| *PIRL5* | ++ | ̶ | + |  | ̶ | ̶ | ̶ | ̶ |  | ̶ | ̶ | ̶ | + | ̶ | ̶ | ̶ | ̶ |
| *PIRL6* | ̶̶ | ̶ | ̶ |  | ̶ | ̶ | ̶ | ̶ |  | ̶ | ̶ | ̶ | + | ++ | ++ | + | + |
| *PIRL7* | + | ̶ | ++ |  | ̶ | ̶ | ̶ | ̶ |  | ̶ | ̶ | ̶ | + | ++ | ++ | ++ | ̶ |
| *PIRL8* | + | ̶ | ++ |  | ++ | ̶ | ̶ | ̶ |  | ̶ | ̶ | ̶ | ̶ | ̶ | ̶ | ̶ | ̶ |
| *PIRL9* | + | ̶ | ++ |  | ̶ | ̶ | ̶ | ̶ |  | ̶ | ̶ | ̶ | ̶ | ̶ | ̶ | ̶ | ̶ |

̶ Not stained

+ Weakly stained

++ Strongly stained
